# Small Cell Neuroendocrine Carcinoma of the Uterine Cervix: A Case Report Highlighting a Rare and Aggressive Tumor

**DOI:** 10.7759/cureus.89439

**Published:** 2025-08-05

**Authors:** Nektarios Ntalakos, Maria Arnaouti, Evdokia Arkoumani

**Affiliations:** 1 Department of Pathology, Saint Savvas Anticancer Hospital of Athens, Athens, GRC

**Keywords:** gynecologic oncology, gynecologic pathology, neuroendocrine carcinoma of cervix, obstetrics and gynecology (ob-gyn), small cell carcinomas

## Abstract

Small cell neuroendocrine carcinoma of the cervix is an uncommon, aggressive tumor that most often affects women in their 40s and is frequently linked to high-risk human papillomavirus (HPV) infection. It is associated with poor prognosis even in early-stage disease. We report the case of a 36-year-old woman with high-risk HPV who presented with abnormal vaginal bleeding. Colposcopy, followed by biopsy and positron-emission tomography computed tomography, confirmed International Federation of Gynecology and Obstetrics stage IIB small cell neuroendocrine cervical carcinoma. The patient underwent external beam radiotherapy and brachytherapy, followed by cisplatin and etoposide chemotherapy. A significant reduction in tumor size was achieved, and interval surgery confirmed residual neuroendocrine carcinoma with extensive vascular invasion. Its diagnosis requires careful histopathologic and immunohistochemical evaluation to differentiate it from morphologic mimics and to exclude metastatic disease. Due to its rarity, there are no standardized treatment protocols, with current approaches favoring individualized, multidisciplinary management. Chemoradiation remains the cornerstone of therapy in locally advanced disease. The potential utility of immunotherapy and targeted agents is currently under investigation. Small cell neuroendocrine carcinoma of the uterine cervix remains a challenging malignancy to diagnose and treat, with early recognition and coordinated multimodal care able to improve outcomes.

## Introduction

This is an uncommon and high-grade malignancy, consisting of small neoplastic cells that exhibit minimal cytoplasm and show features typical of neuroendocrine differentiation [1]. While it can develop anywhere throughout the gynecologic tract, the uterine cervix represents the most frequent site of origin [1]. It constitutes roughly 2% of all cervical carcinomas and can affect individuals across a broad age spectrum (21-87 years), though it typically peaks in their fifth decade [2]. Unlike small cell neuroendocrine carcinomas in other anatomical regions, which tend to arise after menopause, cervical cases often present earlier in life [1]. High-risk human papillomavirus (HPV) infection, especially types 16 and 18, is strongly implicated in its pathogenesis [1]. Compared to other poorly differentiated cervical cancers, small cell neuroendocrine carcinoma is associated with a significantly poorer prognosis [3]. Even in early-stage disease, outcomes are unfavorable, with the five-year survival rates being reported as low as 14% [3]. Nevertheless, select studies have shown improved survival, up to 55%, in patients receiving multimodal therapy [2]. Prognosis is generally more favorable in cases diagnosed at stage I, with smaller tumor size, superficial invasion, chromogranin negativity, and absence of lymph node involvement serving as positive indicators [2].

## Case presentation

A 36-year-old female patient with a known history of high-risk HPV infection presented to the Gynecology Outpatient Clinic with abnormal vaginal bleeding that had persisted for approximately three months. Colposcopic examination revealed a cervical mass, and a cervical biopsy was subsequently performed.

**Figure 1:**
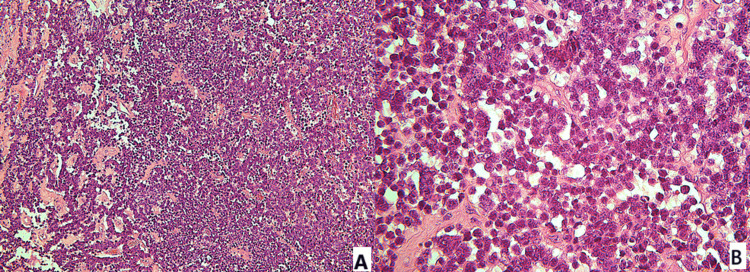
Histopathological imaging of small cell neuroendocrine carcinoma of the uterine cervix. (A) H&E stain (100×). (B) H&E stain (400×) H&E: hematoxylin and eosin

**Figure 2:**
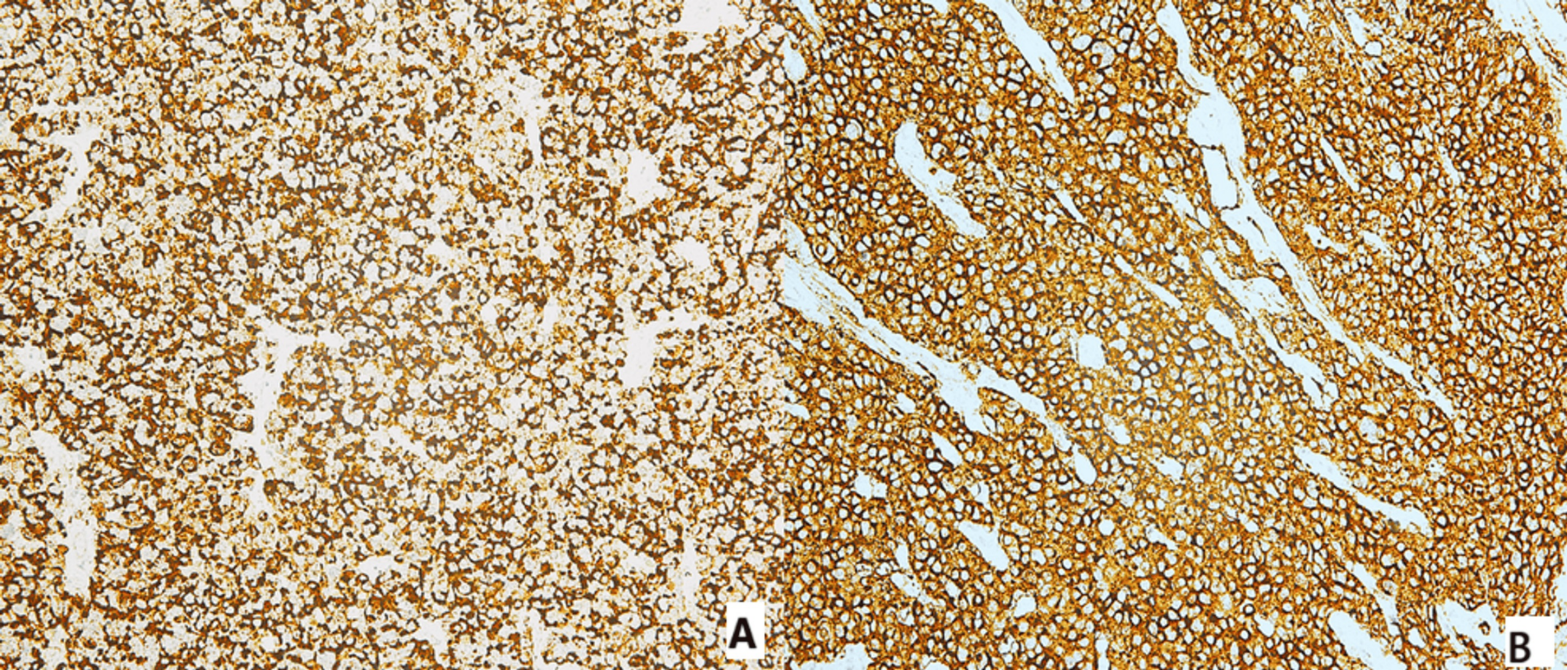
Immunohistochemical analysis of small cell neuroendocrine carcinoma of the uterine cervix. Neoplastic cells positive for (A) chromogranin and (B) synaptophysin

**Figure 3:**
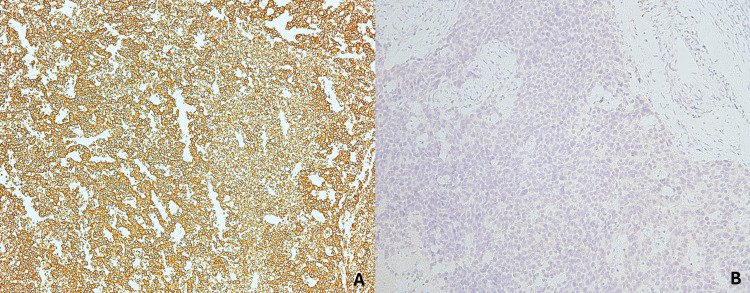
Immunohistochemical analysis of small cell neuroendocrine carcinoma of the uterine cervix. Neoplastic cells (A) positive for cytokeratin AE1/AE3 and (B) negative for p63

Histologically, the tumor was composed of highly atypical cells with scant to indiscernible cytoplasm, ovoid to slightly spindled hyperchromatic nuclei with dispersed chromatin, and inconspicuous nucleoli. Additional findings included nuclear molding, brisk mitotic activity, and crush artifact (Figure 1). Immunohistochemical analysis demonstrated strong positivity for cytokeratin AE1/AE3, chromogranin, and synaptophysin, and negativity for p63, confirming the diagnosis of small cell neuroendocrine carcinoma of the uterine cervix (Figures 2, 3).

Staging was initiated with positron-emission tomography computed tomography (PET-CT) and pelvic MRI, which identified a cervical mass measuring 40 mm in greatest dimension, and corresponding to International Federation of Gynecology and Obstetrics (FIGO) stage IIB. The patient underwent chemoradiotherapy, receiving 25 fractions of external beam radiation therapy, with a total dose of 45 Gy over five weeks, followed by high-dose-rate brachytherapy, with a total dose of 28 Gy over four weeks. This was succeeded with five cycles of cisplatin and etoposide.

Follow-up PET-CT and MRI at three months posttreatment revealed the reduction of tumor size to 22 mm in the greatest dimension. Subsequently, the patient underwent total hysterectomy with bilateral salpingo-oophorectomy, and the specimen was submitted to the Pathology Department for further evaluation. Histopathological examination of the surgical specimen confirmed the diagnosis of residual small cell neuroendocrine carcinoma of the cervix, with extensive vascular invasion also being observed.

## Discussion

Small cell neuroendocrine carcinoma arising within the gynecologic tract is recognized for its highly aggressive clinical behavior, with lymphovascular invasion being observed in a vast majority of cases [3]. Accurate diagnosis necessitates a thorough histopathological evaluation supported by immunohistochemistry, as the tumor must be differentiated from several histologic mimics [2,3]. These include nonkeratinizing squamous cell carcinoma, undifferentiated carcinoma, large cell neuroendocrine carcinoma, adenoid basal carcinoma, lymphoma, and endometrial stromal sarcoma. It is also essential to exclude metastatic origin from the lung [2,3]. The lack of a primary pulmonary lesion on imaging, the presence of in situ carcinoma within the cervix, and the detection of high-risk HPV within the tumor support a primary cervical origin over a pulmonary one [2].

Due to the rarity of small cell neuroendocrine carcinoma of the cervix, there is a lack of robust, evidence-based guidelines to inform treatment decisions [4,5]. As such, therapeutic strategies must often be tailored to individual cases and determined through multidisciplinary collaboration.

For early-stage disease, the most frequently adopted approach involves radical hysterectomy with lymph node dissection, followed by adjuvant chemotherapy, typically a combination of etoposide and cisplatin. In cases of locally advanced disease, a concurrent chemoradiotherapy regimen is generally recommended. Some studies have also explored the use of neoadjuvant chemotherapy followed by radical surgery as an alternative option [4,5].

For metastatic or FIGO stage IVB disease, systemic chemotherapy remains the cornerstone of management. While cisplatin and etoposide have traditionally formed the backbone of its treatment, regimens combining paclitaxel and cisplatin with bevacizumab are increasingly favored as first-line options, particularly when the goal is palliative care and symptom control in patients without contraindications [4,5].

Recent studies have indicated the potential benefit of immunotherapeutic agents, such as PD-1 inhibitors (e.g., nivolumab), as well as targeted therapies like bevacizumab; however, these remain investigational, with limited clinical trial data currently available [5].

Close posttreatment monitoring is essential. Recommended follow-up includes pelvic examinations and imaging (CT or PET/CT) every three to six months during the first two years, then every 6-12 months through year five [4,5]. After five years without recurrence, patients may resume standard annual physical and gynecologic evaluations as part of population-based screening protocols [4,5].

## Conclusions

Neuroendocrine carcinoma of the uterine cervix is an uncommon but aggressive neoplasm, frequently presenting diagnostic and treatment challenges due to its histologic similarity to other malignancies and absence of standardized treatment guidelines. Precise diagnosis depends on comprehensive histopathologic assessment and immunohistochemistry. Although the prognosis is generally unfavorable, this case illustrates how a multimodal treatment can contribute to tumor regression and disease control. Personalized treatment planning through multidisciplinary management is essential. Evolving understanding of its molecular characteristics and the potential role of immunotherapy, warrant further research in order to optimize therapeutic strategies.
